# Editorial: Insights in molecular diagnostics and therapeutics: 2022

**DOI:** 10.3389/fmolb.2023.1209844

**Published:** 2023-05-05

**Authors:** Matteo Becatti, William C. Cho

**Affiliations:** ^1^ Department of Experimental and Clinical Biomedical Sciences “Mario Serio”, University of Firenze, Firenze, Italy; ^2^ Department of Clinical Oncology, Queen Elizabeth Hospital, Kowloon, Hong Kong, China

**Keywords:** molecular diagnostics, precision medicine, biomarkers, systems biology, genetic

Molecular diagnostics and therapeutics have revolutionized the way we manage and treat diseases, from the development of targeted therapies to the identification of genetic mutations. In recent years, this field has seen tremendous advancements. This has been fueled by the increased availability of genomic data, which has allowed researchers and clinicians to better understand the underlying genetic mechanisms of diseases. Precision medicine, also known as personalized medicine, is an emerging approach to healthcare that aims to tailor medical treatments to the individual characteristics of each patient. Rather than relying on a one-size-fits-all approach to treatment, precision medicine takes into account factors such as a patient’s genetics, environment, and lifestyle to deliver more targeted and effective care. This approach has the potential to revolutionize healthcare, providing patients with more precise diagnoses and treatments, reducing healthcare costs, and improving patient outcomes. By understanding the unique genetic makeup of each patient, healthcare providers can develop more personalized treatment plans that are tailored to the specific needs and characteristics of each patient. By tailoring treatments to the individual characteristics of each patient, precision medicine has the potential to improve outcomes and reduce healthcare costs across a wide range of conditions. This Research Topic includes three Original Research articles, four Reviews, and one Mini Review, highlighting the most recent developments in molecular diagnostic and therapeutic approaches that are utilized in precision medicine, as well as their applications in biomarker discovery and validation. Despite its potential benefits, precision medicine also presents some challenges. One of the biggest challenges is the need for accurate and reliable data. In order to develop effective personalized treatments, healthcare providers need access to large amounts of accurate data on patients’ genetics, environment, and lifestyle. This requires significant investment in data Research Topic, storage, and analysis, as well as ensuring patient privacy and confidentiality.

Systems biology is a field of study that combines computational, mathematical, and experimental methods to study complex biological systems at a system level. It aims to understand the relationships between different components of biological systems, such as genes, proteins, and metabolites, and how these components interact to produce specific biological functions. In this Research Topic, using system biology approaches, two original research articles (Huang et al.; Gong et al.; Huang et al.) showed the latest results utilizing bioinformatic tools and public databases to identify novel prognostic and diagnostic biomarkers in glioma, oral squamous cell carcinoma, and rheumatoid arthritis.

Cancer drug development and therapeutic decision-making for individual patients require the identification of new prognostic and predictive biomarkers. However, one of the significant challenges today is to develop biomarkers that can determine disease progression and outcome. In this context, in a review article, Shimukus and Nonaka have explored the various diagnostic criteria for subtyping diffuse large B-cell lymphoma at a molecular level and the genetic alterations implicated in disease progression and outcome. In the same way, through system biology and machine learning approaches, Guhe et al. summarized novel therapeutic target for leishmaniasis treatment whereas Sabirov et al. evaluated the results of recent studies looking for novel biomarkers in neurotrauma, analyzing new methods and protocols to identify diagnostic and prognostic biomolecules.

In recent years, single-cell sequencing technologies have revolutionized our understanding of the complexity of biological systems by allowing the characterization of individual cells at the genomic, transcriptomic, and epigenetic levels. They have a wide range of applications in various fields, including developmental biology, cancer research, neuroscience, and immunology. Single-cell sequencing can help identify different cell types within a complex tissue or organ, detect rare cells, characterize tumors, study neurons, and identify immune cell populations. These technologies have revolutionized the study of biology and medicine by enabling the analysis of individual cells with unprecedented resolution. In this Research Topic, Pregizer et al. reviewed current experimental and computational methods for generation and integration of single cell multi-omic datasets. They focus on opportunities for multi-omic single cell sequencing to augment therapeutic development for kidney disease, including applications for biomarkers, disease stratification, and target identification.

Last but not least, Zhao et al. reviewed the art of tetrazine bioorthogonal chemistry for *in vivo* imaging applications. Bioorthogonal chemistry refers to a set of chemical reactions that can occur inside living systems without interfering with the native biological processes. This concept has emerged as a powerful tool for studying and manipulating biological systems, as it allows scientists to selectively label, visualize, and modify biomolecules such as proteins, nucleic acids, and carbohydrates. Bioorthogonal reactions typically rely on small molecules or chemical groups that do not exist in biological systems or have minimal reactivity with biomolecules. Bioorthogonal chemistry has numerous applications in biomedical research, including the imaging and tracking of cells, the detection of disease biomarkers, and the delivery of drugs or therapeutic agents. Because bioorthogonal reactions can be performed in living systems, they provide a way to study and manipulate biological processes in real time, with minimal perturbation to the system under study.

Overall, the articles published in this Research Topic shed light on the latest updates and advancements in molecular diagnostics and therapeutics, and how they are being utilized in precision medicine. Each report raises important questions and highlights areas that require further scientific investigation. The integration of artificial intelligence, machine learning algorithms, and computational biology presents a novel opportunity to transform big data into actionable information, enabling earlier and more accurate diagnosis and treatment. As we continue to advance our understanding of genomic and metagenomic information, combined with the use of artificial intelligence and machine learning, the field of molecular diagnostics will continue to progress, ultimately leading to improved personalized care for patients. This approach involves combining traditional clinical data with patients’ biological profiles, including various omics-based datasets, to pave the way for a new and exciting era of personalized medicine.

